# Inter-individual differences in heart rate variability are associated with inter-individual differences in mind-reading

**DOI:** 10.1038/s41598-017-11290-1

**Published:** 2017-09-14

**Authors:** Alexander Lischke, Daniela Lemke, Jörg Neubert, Alfons O. Hamm, Martin Lotze

**Affiliations:** 1 Department of Psychology, University of Greifswald, Greifswald, Germany; 2 Functional Imaging Unit, Center of Diagnostic Radiology and Neuroradiology, University of Greifswald, Greifswald, Germany; 3 Helios Hospital, Stralsund, Germany

## Abstract

In the present study, we investigated whether inter-individual differences in vagally-mediated cardiac activity (high frequency heart rate variability, HF-HRV) would be associated with inter-individual differences in mind-reading, a specific aspect of social cognition. To this end, we recorded resting state HF-HRV in 49 individuals before they completed the Reading the Mind in the Eyes Test, a test that required the identification of mental states on basis of subtle facial cues. As expected, inter-individual differences in HF-HRV were associated with inter-individual differences in mental state identification: Individuals with high HF-HRV were more accurate in the identification of positive but not negative states than individuals with low HF-HRV. Individuals with high HF-HRV may, thus, be more sensitive to positive states of others, which may increase the likelihood to detect cues that encourage approach and affiliative behavior in social contexts. Inter-individual differences in mental state identification may, thus, explain why individuals with high HF-HRV have been shown to be more successful in initiating and maintaining social relationships than individuals with low HF-HRV.

## Introduction

The ability to infer others’ thoughts, intentions and feelings on basis of facial cues is crucial for initiating and maintaining social relationships. This ability, which is commonly referred to as mind-reading, a specific aspect of social cognition, has received considerable attention over the past decades^1^. Traditionally, mind-reading has been studied with the Reading the Mind in the Eyes Task (RMET), a social cognition task that requires the identification of mental states on basis of subtle cues provided by the eye region of faces^2^. Mind-reading has been shown to be altered in mental disorders that are characterized by marked deficits in social cognition, like, for example, major depression^3^, borderline personality disorder^4^, autism spectrum disorders^2^ or schizophrenia^5^. Interestingly, reductions in heartbeat variability (HRV), a measure of beat-to-beat variations in heart rate that has been suggested to reflect inter-individual differences in social cognition^6^, has also been reported in these disorders^7, 8^. Inter-individual differences in mind-reading may, thus, be associated with inter-individual differences in HRV. Accordingly, it has recently been reported that individuals with high HRV are more accurate in mental state identification than individuals with low HRV^9^. Although these findings indicate that inter-individual differences in HRV and mind-reading are, in fact, associated with one another, it remains open whether individuals with high and low HRV show valence-unspecific or valence-specific differences in mental state identification. Of note, valence-specific differences in mental state identification are known to be associated with differences in social behavior^1^ and differences in social behavior have already been observed in individuals with high and low HRV^10–12^. We, thus, assumed that individuals with high and low HRV would show valence-specific rather than valence-unspecific differences in mental state identification. We also assumed that differences in mental state identification would emerge during the processing of positive states that encourage approach rather than during the processing of negative states that encourage avoidance because differences in the processing of approach-related cues as well as in the initiation and maintenance of approach behavior have already been reported in individuals with high and low HR^10–12^. To test these assumptions, we investigated whether inter-individual differences in HRV would be associated with inter-individual differences regarding the identification of positive and negative states in the RMET.

## Method

### Participants

Before we recruited our participants, we performed a power analysis with G*Power 3^13^ to determine the number of participants that we needed to detect meaningful associations between inter-individual differences in HRV and inter-individual differences in mental state identification. Previous studies investigating associations between inter-individual differences in HRV and inter-individual differences in social cognition revealed medium sized correlations (mean *r* = 0.35) between HRV and task performance^9, 14^. These studies reported positive correlations indicating that task performance increased with increasing HRV. To detect a similar directed correlation (*r* = 0.35) with sufficient power (1-*β* = 80) and a conventional significance value (*α =* 0.05), 46 participants would be required at minimum. Taking potential drop-outs into account, we recruited 49 participants for our study from the university campus using flyers. In order to be included in the study, participants had to be aged between 18 and 35 years and to be native German speakers. Following established guidelines^15^, participants suffering from mental or physical health conditions and participants using other medication than oral contraceptives were excluded. Inclusion and exclusion of participants was determined on basis of an in-house interview assessing participants’ physical and mental health as well as participants’ utilization of physical and mental health care. The in-house interview was conducted by a trained graduate student in psychology (D.L.) under supervision of a clinical psychologist (A.L.), a clinical psychotherapist (A.O.H.) and a medical doctor (M.L.). Of the 49 participants that were included in the study, 7 did not provide valid data due to equipment dysfunction. Consequently, the data of 42 participants, 21 males and 21 females, were considered in the analyses (see Table 1).

**Table 1:** Participant characteristics.

	*M (SD)*	95% CI
Age	23.03 (3.56)	[21.98, 24.40]
Sex (m/f)	21/21	
**Heart rate variability (HRV)**		
HF-HRV (ms^2^)	1003.48 (1248.57)	[614.40, 1392.56]
Log HF-HRV (ms^﻿﻿2^)	2.78 (0.45)	[2.64, 2.92]
Psychopathology (BSI-18-GSI)	0.40 (0.28)	[0.31, 0.49]
**Mind-reading (RMET)**		
Positive states^a^	0.76 (0.16)	[0.71, 0.81]
Negative states^b^	0.66 (0.16)	[0.61, 0.71]
Neutral states^c^	0.67 (0.13)	[0.63. 0.71]

*Note*. m = male, f = female, HF-HRV = high frequency heart rate variability, Log HF-HRV = log transformed high frequency heart rate variability, BSI-18-GSI = Brief Symptom Inventory - 18 - Global Severity Index^17^, RMET = Reading the Mind in the Eyes Test^2, 22^. ^a^Percentage of correctly identified states for eye regions with the following item numbers^24^: 1, 6, 16, 20, 21, 25, 30, 31. ^b^Percentage of correctly identified states for eye regions with the following item numbers^24^: 2, 5, 11, 14, 17, 22, 23, 26, 27, 34, 35, 36. ^c^Percentage of correctly identified states for eye regions with the following item numbers^24^: 3, 4, 7, 8, 9, 10, 12, 13, 15, 18, 19, 24, 28, 29, 32, 33.

All participants provided written-informed consent before taking part in the study and received 10 € after completion of the study. The study was approved by the ethics committee of the German Psychological Society (DGPs) and carried out in accordance with the Declaration of Helsinki.

### Procedure

After arriving at the laboratory, participants were asked to use the bathroom to control for the effects of bladder filling and gastric distension on HRV^16^. Participants were then seated in a comfortable chair in a sound-attenuated room (temperature: 22–23 °C). Following an acclimatization period of 5 min, a 5 min lasting electrocardiogram (ECG) was recorded with an Eindhoven Lead II set-up. In line with previous studies^9, 14^, participants were asked to breathe spontaneously and to keep their eyes open during the recording. Thereafter, participants performed the RMET^2^ and completed the Brief Symptom Inventory 18^17^, a global measure of psychopathology.

### Heart rate variability

An Eindhoven Lead II setup with two standard, electrolyte filled, Ag/AgCl electrodes (8 mm; Marquette Hellige, Freiburg, Germany) was used to record an ECG for 5 minutes. Online, the ECG signal was filtered with an 8 to 13 Hz band-pass filter, amplified with the factor 2000 and sampled at rate of 1000 Hz using a Coulbourn S75-01 system (Coulbourn Instruments, Whitehall, PA, USA). Offline, the ECG signal was down sampled to 400 Hz and further processed with ANSLAB 2.4^18^. Using ANSLAB 2.4, the ECG signal was visually inspected for artifacts (e.g., movement artifacts, physiological artifacts). Whenever necessary, the ECG signal was manually corrected via beat replacement instead of beat removal as recommend in recent guidelines^15^. Finally, Kubios HRV 2.2^19^ was used to determine HRV following established guidelines^20^. Similarly as in previous studies^9, 14^, high frequency HRV (HF-HRV; 0.15–0.4 Hz) was estimated on basis of a Fast Fourier Transformation (Welch’s periodogram: 256 s window with 50% overlap). In contrast to other indices of HRV, HF-HRV reflects parasympathetic rather than sympathetic induced changes in cardiac activity that are mediated by the vagus nerve^21^.

### Reading the Mind in the Eyes Test

The RMET, which is an established social cognition task, comprised 36 black and white pictures of eye regions expressing various mental states, including positive, negative and neutral ones^2^. The eye regions were shown in random order to participants on a computer screen (size: 20 inch, resolution: 1024 × 728 pixel; viewing distance: 55 cm; black background). Each eye region was shown together with four labels describing a particular mental state (3 distractors, 1 target). Participants had to indicate the label that best described the mental state by pressing a corresponding button as fast as possible. Following the approach of previous studies^22, 23^ using a similar RMET version to investigate mental state identification, participants’ responses were analyzed separately for positive, negative and neutral states on basis of a validated classification scheme^24^. After determining the raw percentages of correctly identified positive, negative and neutral states, relative percentages of correctly identified positive and negative states were determined by subtracting the raw percentage of correctly identified neutral states from the raw percentages of correctly recognized positive and negative states, respectively. This subtraction method^25^, which is common in psychological studies^26–29^, was necessary to determine whether differences in mental state identification were specific for positive or negative as compared to neutral states. It also allowed the control of differences in mental state identification that were associated with the well-known ambiguity of neutral states^30–32^.

### Statistical Analysis

All statistical analyses were conducted using SPSS 22 (SPSS Inc., Chicago, IL, USA). To investigate whether inter-individual differences in HRV would be associated with inter-individual differences in the identification of positive and negative states in the RMET, three sets of analyses were run. In each set of analyses, sex, age and psychopathology as assessed with the Brief Symptom Inventory 18^17^ were used as covariates in partial correlations to control for factors that are known to affect HF-HRV^15^. In the first set of analyses, partial correlations were computed between HF-HRV and the difference between the percentage of correctly identified positive relative to neutral states and the percentage of correctly identified negative relative to neutral states. In the second set of analyses, partial correlations were computed between HF-HRV and the percentage of correctly identified positive relative to neutral states as well as between HF-HRV and the percentage of negative relative to neutral states. In the third set of analyses, the aforementioned partial correlations were compared with one another using Steiger’s Z-Test^33^. Prior to all analyses, HF-HRV was log transformed (log 10) to account for deviations from normality distribution. The significance level for all analyses was set at *p* < 0.05 one-sided due to the hypothesis-driven nature of the respective analyses. To facilitate the interpretation of the respective findings, 95% confidence intervals (CI) and effect size measures (*r, q*) are reported^34^. Small effect sizes are indicated by *r* = 0.1 or *q* = 0.1, medium effect sizes are indicated by *r* = 0.3 or *q* = 0.3 and large effect sizes are indicated by *r* = 0.5 or *q* = 0.5.

### Data availability

The data that was used for the aforementioned analyses are available from the corresponding author on reasonable request.

## Results

**Figure 1:**
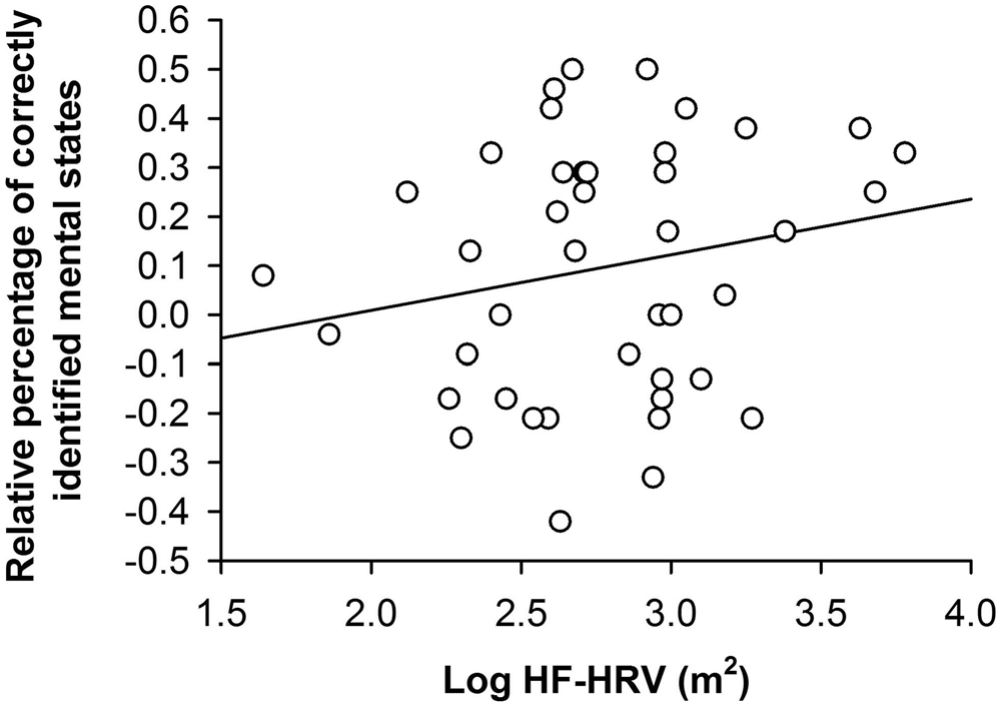
Correlation between HF-HRV and the difference between the relative percentages of correctly identified positive and negative states in the RMET^2^.

The first set of analyses indicated that HF-HRV correlated with the difference between the relative percentages of correctly identified positive and negative states (*r*(37) = 0.295, *p* = 0.034; 95% CI [−0.01, 0.55]; see Fig. 1). Although the size and direction of the correlation suggested that HF-HRV correlated more with the relative percentage of correctly identified positive than negative states, there was some ambiguity associated with the estimation of the corresponding correlation coefficient as indicated by the size of the respective confidence interval. However, the second set of analyses also indicated that HF-HRV correlated with the relative percentage of correctly identified positive states (*r*(37) = 0.401, *p* = 0.006; 95% CI [0.11, 0.63]) rather than with the relative percentage of correctly identified negative states (*r*(37) = −0.139, *p* = 0.199; 95% CI [−0.43, 0.17]). The third set of analyses, which involved a formal comparison of the respective correlation coefficients, confirmed that HF-HRV correlated with the relative percentage of correctly identified positive but not negative states (*z* = 2.223, *p* = 0.013, *q* = 0.565). Moreover, using raw instead of relative percentages of correctly identified states in the aforementioned analyses yielded a similar pattern of correlation coefficients (see Supplementary Results). Notably, the correlation coefficients describing the correlation between HF-HRV and the percentage of correctly identified positive states corresponded to medium or large effect sizes, whereas the correlation coefficients describing the correlation between HF-HRV and the percentage of correctly identified negative states corresponded to small effect sizes. HF-HRV was, thus, substantially and robustly correlated with the percentage of correctly identified positive states in a series of complementary analyses.

## Discussion

In the present study, we investigated whether inter-individual differences in HRV would be associated with inter-individual differences regarding the identification of positive and negative states in the RMET. In accordance with our assumptions, we found an association between inter-individual differences in HRV and inter-individual differences in mental state identification during the processing of positive but not negative states. Individuals with high HRV were more accurate in the identification of positive but not negative states than individuals with low HRV, indicating an increased identification of positive states with increasing HRV.

On basis of these findings, it may cautiously be speculated that inter-individual differences in HRV may be associated with inter-individual differences in mental state identification as well as with inter-individual differences in approach and avoidance behavior. Individuals with high HRV may be more sensitive to positive states of others’ than individuals with low HRV. Individuals with high HRV may, thus, be more likely to detect cues that encourage approach and affiliation behavior, like, for example, a subtle smile, than individuals with low HRV. As a consequence, individuals with high HRV may be more likely to approach others than individuals with low HRV. Approaching others that are in a positive state may increase the likelihood of positive interactions, which may lead to a plethora of positive experiences in the approaching individual as well as in the approached individual. Accordingly, it has been shown that individuals with high HRV are more successful in initiating and maintaining social relationships than individuals with low HRV^10–12^, which has been found to be associated with feelings of well-being and connectedness^10, 35, 36^. In this respect it is interesting to note that individuals with low HRV have often been found among individuals with mental disorders that a characterized by marked deficits in social cognition and social interaction^7, 8^. Inter-individual differences in HRV may, thus, account for inter-individual differences in social cognition and social behavior in healthy as well as in mentally-disordered individuals.

Regarding the neurobiological mechanisms mediating the association between inter-individual differences in HRV and inter-individual differences in mental state identification, it is noteworthy that a network of prefrontal and temporal brain regions has been shown to be engaged during mental state identification^37–41^. In particular, the prefrontal cortex and the amygdala have been reported to be crucial for the identification of mental states^38, 41^. Accordingly, it has been found that individuals with functional and structural alterations in these brain regions show alterations in mental state identification^4, 37, 38, 41–43^, presumably because the interplay between the prefrontal cortex and the amygdala is disturbed in these individuals. Of note, inter-individual differences regarding functional and structural alterations in the prefrontal cortex and in the amygdala have been shown to be associated with inter-individual differences in HRV^44, 45^. Moreover, inter-individual differences in HRV have even been considered to be a direct indicator of inter-individual differences regarding the interplay of the prefrontal cortex and the amygdala^46–48^. More precisely, an increase in HRV is thought to reflect an increase in prefrontal activity as well as an increase in prefrontal-amygdala connectivity. It may, thus, be possible that individuals with high HRV were more accurate in mental state identification than individuals with low HRV because they showed more prefrontal activity and prefrontal-amygdala connectivity during the processing of mental states.

Overall, our findings suggest that inter-individual differences in HRV may be associated with inter-individual differences in the identification of mental states, presumably due to inter-individual differences in prefrontal activity and prefrontal-amygdala connectivity during the processing of mental states. An increase in prefrontal activity and prefrontal-amygdala connectivity, which is reflected by an increase in HRV, may lead to an increased processing of positive as compared to negative states during encounters with others, thereby facilitating approach and affiliative behavior in social contexts. Recent theories regarding the neurobiological mechanisms mediating the association between inter-individual differences in HRV and inter-individual differences in social cognition and social interaction appear to be consistent with our assumptions^46–48^. Moreover, our assumptions are also consistent with recent findings regarding inter-individual differences in HRV and inter-individual differences in social cognition and social interaction^9, 10, 12, 35, 36^.

However, our assumptions should be treated with caution for several reasons. First and foremost, we employed a correlational study design that precludes any causal inferences about the direction of causality between inter-individual differences in HRV and inter-individual differences in mental state identification. Second, we assessed inter-individual differences in HRV and inter-individual differences in mental state identification but not inter-individual differences in approach and avoidance behavior. It is, thus, unclear whether inter-individual differences in HRV are in fact similarly associated with inter-individual differences in social behavior as with inter-individual differences in social cognition. Third, we did not assess inter-individual differences in prefrontal activity and prefrontal-amygdala connectivity, leaving open whether these differences in fact mediate the association between inter-individual differences in HRV and inter-individual differences in social cognition or social behavior. Fourth, our study sample consisted of healthy young adults, indicating that our assumptions regarding the association between inter-individual differences in HRV and inter-individual differences in social cognition and social behavior cannot be generalized to other study samples. Consequently, experimental rather than correlations studies are warranted that further test our assumptions, preferably by recording brain activity and cardiac activity in concert during tasks that affect both social cognition and social behavior in large samples of healthy and mentally-disordered individuals with different age ranges.

## Electronic supplementary material

Supplementary Info
