# Supplementary material for: Inter-individual differences in heart rate variability are associated with inter-individual differences in mind-reading
Source: Sci Rep. 2017 Sep 14;7:11557. doi: 10.1038/s41598-017-11290-1 (PMC5599679; doi:10.1038/s41598-017-11290-1)
Supplement: Supplementary file 1 — Supplementary Info [file 41598_2017_11290_MOESM1_ESM.doc]

**Inter-individual differences in heart rate variability are associated with inter-individual differences in mind-reading**

Alexander Lischke^1,2^, Ph.D.; Daniela Lemke^3^; Jörg Neubert^1^; Alfons O. Hamm^1*^, Ph.D.; Martin Lotze^2*^, M.D.

**Supplementary Results**

The first set of analyses indicated that HF-HRV correlated with the difference between the raw percentages of correctly identified positive and negative states (*r*(37) = .295, *p* = .034; 95% CI [-.01, .55]), suggesting that HF-HRV correlated more with the raw percentage of correctly identified positive than negative states. The second set of analyses also indicated that HF-HRV correlated with the raw percentage of correctly identified positive states (*r*(37) = .379, *p* = .009; 95% CI [.09, .61]) rather than with raw percentage of correctly identified negative states (*r*(37) = -.084, *p* = .305; 95% CI [-.38, .23]). The third set of analyses, which involved a formal comparison of the aforementioned correlation coefficients, confirmed that HF-HRV correlated with the raw percentage of correctly identified positive but not negative states (*z* = 2.223, *p* = .013, *q* = 0.565). An inspection of the respective correlation coefficients revealed that the correlation coefficient describing the correlation between HF-HRV and the raw percentage of correctly identified positive states corresponded to a medium or large effect size and that the correlation coefficient describing the correlation between HF-HRV and the raw percentage of correctly identified negative states corresponded to a small effect size. HF-HRV was, thus, substantially correlated with the raw percentage of correctly identified positive states.
